# Development and validation of diagnostic and activity-assessing models for relapsing polychondritis based on laboratory parameters

**DOI:** 10.3389/fimmu.2023.1274677

**Published:** 2023-10-03

**Authors:** Yongmei Liu, Linlin Cheng, Mengzhu Zhao, Haoting Zhan, Xiaomeng Li, Yuan Huang, Haolong Li, Yong Hou, Yongzhe Li

**Affiliations:** ^1^Department of Clinical Laboratory, State Key Laboratory of Complex, Severe and Rare Diseases, Peking Union Medical College Hospital, Peking Union Medical College and Chinese Academy of Medical Sciences, Beijing, China; ^2^Department of Rheumatology and Clinical Immunology, State Key Laboratory of Complex Severe and Rare Diseases, Peking Union Medical College Hospital, Chinese Academy of Medical Sciences and Peking Union Medical College. National Clinical Research Center for Dermatologic and Immunologic Diseases (NCRC-DID), Ministry of Science and Technology. Key Laboratory of Rheumatology and Clinical Immunology, Ministry of Education, Beijing, China; ^3^Department of Rheumatology, The First Affiliated Hospital, Zhejiang University School of Medicine, Hangzhou, China; ^4^Department of Clinical Laboratory, Peking University People’s Hospital, Beijing, China

**Keywords:** relapsing polychondritis, disease activity, laboratory markers, monocyte, NLR, CAR, lymphocyte subsets, prediction model

## Abstract

**Background:**

Relapsing polychondritis (RP) as a rare autoimmune disease is characterized by recurrent inflammation of the organs containing cartilage. Currently, no biomarkers have been integrated into clinical practice. This study aimed to construct and evaluate models based on laboratory parameters to aid in RP diagnosis, assess activity assessment, and explore associations with the pathological process.

**Methods:**

RP patients and healthy controls (HCs) were recruited at the Peking Union Medical College Hospital from July 2017 to July 2023. Clinical data including Relapsing Polychondritis Disease Activity Index (RPDAI) score and laboratory tests were collected. Differences in laboratory data between RP patients and HCs and active and inactive patients were analyzed.

**Results:**

The discovery cohort (cohort 1) consisted of 78 RP patients and 94 HCs. A model based on monocyte counts and neutrophil to lymphocyte ratio (NLR) could effectively distinguish RP patients from HCs with an AUC of 0.845. Active RP patients exhibited increased erythrocyte sedimentation rate, complement 3, platelet to lymphocyte ratio (PLR), NLR, and C-reactive protein to albumin ratio (CAR) compared with stable patients, which were also positively correlated with RPDAI. Notably, CAR emerged as an independent risk factor of disease activity (OR = 4.422) and could identify active patients with an AUC of 0.758. To confirm the reliability and stability of the aforementioned models, a replication cohort (cohort 2) was enrolled, including 79 RP patients and 94 HCs. The monocyte-combined NLR and CAR showed a sensitivity of 0.886 and 0.577 and a specificity of 0.830 and 0.833 in RP diagnosis and activity prediction, respectively. Furthermore, lower natural killer cell levels in RP patients and higher B-cell levels in active patients may contribute to elucidating the pathological mechanisms of disease occurrence and exacerbation.

**Conclusions:**

The utilization of laboratory parameters provides cost-effective and valuable markers that can assist in RP diagnosis, identify disease activity, and elucidate pathogenic mechanisms.

## Background

Relapsing polychondritis (RP) is a rare multisystemic autoinflammatory disease with an incidence ranging from 0.71 to 4.5 per million population (1–5). The typical clinical manifestations encompass recurrent inflammation of the ear, nose, throat, trachea, and other organs, displaying significant individual heterogeneity (6). Biomarkers for RP discovered in previous studies have not been applied clinically due to limited sensitivity or specificity (7–9).

Existing evidence strongly supports the utility of diverse laboratory tests in various autoimmune diseases. Specifically, monocyte counts, mean corpuscular hemoglobin (MCH) and mean corpuscular hemoglobin concentration (MCHC), neutrophil to lymphocyte ratio (NLR), and lactate dehydrogenase (LDH) level can be used to assess cardiovascular risk in systemic lupus erythematosus (SLE) (10), Behcet’s disease severity (11), the prognosis of immunoglobulin A nephropathy (12), or neurological involvement of adult-onset Still’s disease (13), respectively. Additionally, the C-reactive protein to albumin ratio (CAR), NLR, and platelet to lymphocyte ratio (PLR) were positively correlated with the Relapsing Polychondritis Disease Activity Index (RPDAI) (14). However, the sensitivity and specificity of three new inflammatory markers in distinguishing active patients were not evaluated, nor was the potential of laboratory parameters in identifying RP occurrence. Also, no hematological biomarkers have been integrated into the diagnostic criteria for RP, resulting in diagnosis delays (5). Thus, it is critical to excavate cost-effective and convenient indices for RP in routine clinical practice.

Due to the unclear etiology and pathogenesis of RP, diagnosis and disease activity assessment are based on several clinical criteria (15–18), which primarily rely on the clinician’s experience. However, it is not a quantifiable standard, having limited objectivity. Abundant laboratory data, including hematological and lymphocyte subset analysis, are readily available in clinical practice. Therefore, this study aims to evaluate the effectiveness of models utilizing routine laboratory parameters to identify RP patients, predict disease recurrence, and investigate pathological correlation.

## Materials and methods

### Patients

A total of 157 patients with RP were recruited from Peking Union Medical College Hospital (PUMCH) from July 2017 to July 2023. RP diagnosis was based on the criteria of McAdam (16) or Damiani and Levine (17) or Michet (15). RP patients who also had other autoimmune diseases, tumors, hematological disorders, or immunodeficiency diseases were excluded. Active patients were defined as those with the appearance of new signs or symptoms, recurrence or aggravation of signs or symptoms of existing disease, or worsening of organ involvement assessed via imaging in the previous 28 days. The RPDAI score (18) was applied for all RP patients, and it was calculated independently by two experienced rheumatologists according to clinical manifestations during the previous 28 days. Disagreements between two rheumatologists were resolved by a third senior rheumatologist who made the final decision.

The clinical and laboratory parameter data such as routine blood tests, erythrocyte sedimentation rate (ESR), CRP, and biochemical indices of the selected patients were collected.

To form a control group, 188 gender- and age-matched healthy controls (HCs) were recruited (**Supplementary Table 1**). All participants were randomly divided into two cohorts in a 1:1 ratio utilizing the “complete_ra” function of the “randomizr” packages in R software. The discovery cohort (named cohort 1) consisting of 78 RP patients and 94 HCs was used for identifying valuable laboratory markers, while the replication cohort (named cohort 2) comprising 79 RP patients and 94 HCs was used for verification.

This study was approved by the Institutional Review Board of PUMCH with informed consent acquired from all the enrolled participants (I-23PJ540).

### Lymphocyte subset measurement

Fresh blood samples from 42 RP patients and 42 HCs were collected for the detection of the lymphocyte subsets. The exact antibody clones (Beckman Coulter, CA, USA) that were used for flow cytometric analysis are as follows: PC7 anti-CD3 (737657), anti-CD3-FITC/(CD16 + 56)-PE (A07735), FITC anti-CD45 (A07782), PE anti-CD4 (A07751), PE anti-CD19 (A07769), and PC5 anti-CD8 (A07758). The cell populations were as follows: B lymphocytes (CD45^+^CD19^+^), CD3^+^T lymphocytes (CD45^+^CD3^+^), CD4^+^T lymphocytes (CD45^+^CD3^+^CD4^+^), CD8^+^T lymphocytes (CD45^+^CD3^+^CD8^+^), and natural killer (NK) cells (CD3^-^CD16^+^CD56^+^).

Lymphocyte subsets were detected in three tubes based on T lymphocytes, B lymphocytes, and NK cells. According to the manufacturer’s instruction, the corresponding antibody reagent and 50 μl of whole blood were first added into a tube and incubated at room temperature in the dark for 20 min. Then, 450 μl of hemolysin (A11895, Beckman Coulter, CA, USA) was added to each tube, and after incubation at room temperature in the dark for 15 min, the percentages of the lymphocyte subsets were detected on the Beckman NAVIOS analyzer (Beckman Coulter, CA, USA). The absolute values of total B cells, NK cells, T cells, and T-cell subsets were calculated from the absolute values of lymphocytes counted by the hematology analyzer.

### Statistical analysis

Statistical analysis was performed using R version 4.1.3 software, IBM SPSS Statistics version 26.0 (IBM Corp., USA), and Prism 8.0 (GraphPad, San Diego, CA, USA). Independent sample *t*-test and the Wilcoxon rank-sum test were respectively applied to analyze normally and non-normally distributed data. For categorical variables, the *χ*^2^ test was performed. Correlation analysis of the non-normally distributed data was done by Spearman’s correlation coefficients. *p <*0.05 was considered statistically significant.

## Results

### Building laboratory index models in cohort 1

#### Demographic and clinical features of patients with RP

In cohort 1, there were 35 active patients and 43 inactive patients with no difference in age (43.91 vs. 43.63 years, *p* = 0.928) and gender ratio (male to female, 0.94:1 vs. 0.54:1, *p* = 0.254). The RPDAI score of the active patients was significantly higher than that of the inactive patients (19.77 vs. 0.33, *p* < 0.001). The corresponding clinical features of RP patients enrolled in this study are displayed in **Supplementary Table 2**.

#### Monocyte counts and NLR increased in RP patients

Based on the findings from cohort 1, various parameters were observed to be significantly altered in RP patients compared with HCs. RP patients exhibited higher levels of CRP, white blood cell (WBC), neutrophil, monocyte, platelet, NLR, and PLR, whereas albumin, the percentage of lymphocytes, red blood cell count, hemoglobin, hematocrit, MCHC, and lymphocyte to monocyte ratio (LMR) were lower (all *p* < 0.05, **Table 1**).

**Table 1:** Laboratory findings between RP patients and HCs in cohort 1.

Laboratory parameters	RP (*n* = 78)	HC (*n* = 94)	*p*-value	Laboratory parameters	RP (*n* = 78)	HC (*n* = 94)	*p*-value
Inflammatory parameters							
CRP (mg/L)	2.17 (11.39)	0.63 (0.60)	<0.001	Albumin (g/L)	43.00 (6.00)	45.00 (3.00)	<0.001
WBC-related parameters							
WBC (×10^9^/L)	9.21 (4.83)	6.08 (1.72)	<0.001				
Neutrophil (×10^9^/L)	6.02 (3.97)	3.45 (1.19)	<0.001	Neutrophil (%)^a^	67.34 (10.85)	57.15 (5.79)	<0.001
Monocyte (×10^9^/L)	0.46 (0.29)	0.31 (0.11)	<0.001	Monocyte (%)	5.55 (1.95)	5.30 (1.28)	0.190
Lymphocyte (×10^9^/L)	1.86 (1.11)	1.89 (0.69)	0.651	Lymphocyte (%)	24.00 (14.55)	33.25 (7.33)	<0.001
Basophil (×10^9^/L)	0.03 (0.02)	0.03 (0.02)	0.122	Basophil (%)	0.30 (0.30)	0.50 (0.30)	<0.001
Eosinophil (×10^9^/L)	0.04 (0.06)	0.11 (0.10)	<0.001	Eosinophil (%)	0.60 (0.78)	1.90 (1.20)	<0.00
LMR	4.38 (3.04)	6.19 (2.58)	<0.001	NLR	2.82 (2.33)	1.70 (0.58)	<0.001
Platelet-related parameters							
Platelet (×10^9^/L)	255.5 (125)	217 (6)	<0.001	PLR	135.5 (107.16)	113.12 (44.34)	0.011
RBC-related parameters							
RBC (×10^12^/L)	4.50 (0.62)	4.72 (0.61)	0.003	Hemoglobin (g/L)^a^	134.12 (18.25)	142.16 (11.66)	0.001
Hematocrit (%)^a^	40.01 (4.58)	41.73 (3.19)	0.006	MCV (fl)	89.00 (8.60)	88.40 (3.78)	0.333
MCH (pg)	30.1 (3.93)	30.1 (1.48)	0.573	MCHC (g/L)	334.5 (17.75)	342 (11)	<0.001

RP, relapsing polychondritis; HC, healthy control; CRP, C-reactive protein; WBC, white blood cell; LMR, lymphocyte to monocyte ratio; NLR, neutrophil to lymphocyte ratio; PLR, platelet to lymphocyte ratio; RBC, bed blood cell; MCV, mean corpuscular volume; MCH, mean corpuscular hemoglobin; MCHC, mean corpuscular hemoglobin concentration.

a Represents normally distributed data, expressed as mean (SD). Others are non-normally distributed data, expressed as median (IQR), IQR = Q3–Q1.

**Figure 1:**
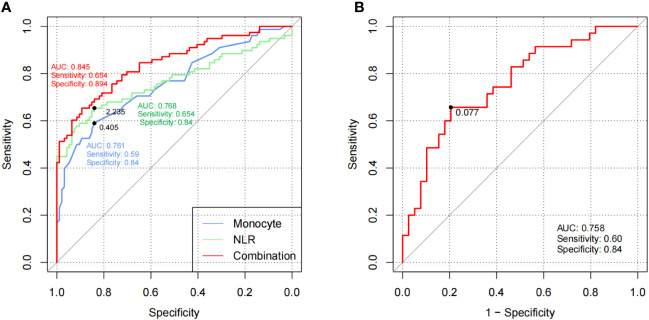
The clinical efficiency of laboratory parameters in discovery cohort 1. **(A)** ROC curves of monocytes and NLR for RP versus HCs in cohort 1. **(B)** ROC curves of CAR for active RP versus inactive RP in cohort 1. The black plots in the curves represented the cutoff values. RP, relapsing polychondritis; NLR, neutrophil to lymphocyte ratio; HCs, healthy controls; ROC, receiver operating characteristic; AUC, area under the ROC curve.

Among the statistically significant indicators, the levels of monocyte, neutrophil, platelet, and NLR were identified as risk factors for RP occurrence through univariate regression analysis. Multivariate regression analysis revealed that high levels of monocyte count [odds ratio (OR) = 1.800, 95% confidence interval (CI) = 1.186–2.732; *p* = 0.006] and NLR (OR = 2.314, 95% CI = 1.261–4.246; *p* = 0.007) significantly enhanced the risk of RP (**Supplementary Table 3**). The AUC, sensitivity, and specificity of monocyte counts (cutoff value = 0.405 × 10^9^/L) were 0.761, 59%, and 84% and those of NLR (cutoff value = 2.235) were 0.768, 65.4%, and 84%, respectively. After the combined application, the diagnostic performance increased to 0.845, 65.4%, and 89.4%, respectively (**Figure 1A**).

#### CAR emerged as a risk factor for RP activity

Compared with patients at the inactive stage in cohort 1, CRP, ESR, neutrophil counts and percentage, IL-6, platelet, complement 3 (C3), PLR, NLR, and CAR were all raised in patients with RP at the active stage, while albumin and lymphocyte percentage were reduced (all *p* < 0.001, **Table 2**).

**Table 2:** Laboratory characteristics of patients with RP at the active and inactive stages in cohort 1.

Laboratory parameters	Active (*n* = 35)	Inactive (*n* = 43)	*p*-value	Laboratory parameters	Active (*n* = 35)	Inactive (*n* = 43)	*p*-value
Inflammatory parameters/cytokines							
CRP (mg/L)	8.22 (25.98)	1.05 (2.43)	<0.001	ESR (mm/h)	16 (37)	6 (12)	<0.001
Albumin (g/L)^a^	40.31 (4.23)	44.71 (3.54)	<0.001	CAR (10^−3^)	0.19 (0.70)	0.02 (0.05)	<0.001
IL-6 (pg/ml)	5.90 (14.63)	2.20 (2.98)	0.034	C3 (g/L)	1.36 (0.31)	1.03 (0.29)	0.010
WBC-related parameters							
WBC (×10^9^/L)	9.76 (4.50)	8.05 (4.33)	0.057				
Neutrophil (×10^9^/L)	6.73 (3.16)	5.00 (3.34)	0.024	Neutrophil (%)^a^	70.45 (9.85)	64.81 (11.08)	0.021
Monocyte (×10^9^/L)^a^	0.51 (0.22)	0.45 (0.14)	0.118	Monocyte (%)^a^	5.51 (1.74)	5.87 (1.74)	0.369
Lymphocyte (×10^9^/L)	1.76 (1.23)	1.94 (0.83)	0.491	Lymphocyte (%)^a^	22.32 (8.70)	27.73 (10.32)	0.016
Basophil (×10^9^/L)	0.03 (0.03)	0.03 (0.02)	0.109	Basophil (%)	0.20 (0.40)	0.40 (0.30)	0.007
Eosinophil (×10^9^/L)	0.04 (0.04)	0.05 (0.07)	0.162	Eosinophil (%)	0.40 (0.55)	0.90 (0.90)	0.064
NLR	3.50 (2.58)	2.46 (1.57)	0.013	LMR	3.97 (2.80)	4.54 (2.83)	0.170
Platelet-related parameters							
Platelet (×10^9^/L)	305 (140.5)	235 (83.5)	0.002	PLR	178.53 (132.01)	115.79 (88.36)	0.020

RP, relapsing polychondritis; HC, healthy control; CRP, C-reactive protein; CAR, CRP to albumin ratio; ESR, erythrocyte sedimentation rate; IL, interleukin. TNF-α, tumor necrosis factor α; WBC, white blood cell; LMR, lymphocyte to monocyte ratio; NLR, neutrophil to lymphocyte ratio; PLR, platelet to lymphocyte ratio; C3, complement 3.

a Represents normally distributed data, expressed as mean (SD). Others are non-normally distributed data, expressed as median (IQR), IQR = Q3–Q1.

CAR, NLR, and PLR were positively associated with RPDAI score. Additionally, ESR (*p* < 0.001), CRP (*p* < 0.001), C3 (*p* = 0.025), and IL-6 (*p* = 0.036) also exhibited positive correlation with disease activity. The indices associated with the RPDAI score are shown in **Supplementary Table 4**.

Univariate regression analysis showed that CAR and PLR were the independent risk factors of disease activity. Multivariate regression analysis confirmed the significance of CAR (OR = 4.422, 95% CI = 1.111–17.605; *p* = 0.035) for indicating the risk of RP activity and recurrence (**Supplementary Table 5**). The cutoff value of CAR was 0.077 × 10^−3^, with an AUC of 0.758, sensitivity of 60%, and specificity of 84% (**Figure 1B**).

### Verification of laboratory parameter models for RP diagnosis and activity assessment

No significant differences in age (48.14 vs. 49.90 years, *p* = 0.571) and gender (male to female ratio, 0.56:1 vs. 0.96:1, *p* = 0.344) were observed between 28 active RP patients and 51 inactive patients of cohort 2. The active patients had higher RPDAI scores than the inactive patients (17.29 vs. 0, *p* < 0.001) (**Supplementary Table 2**).

**Figure 2:**
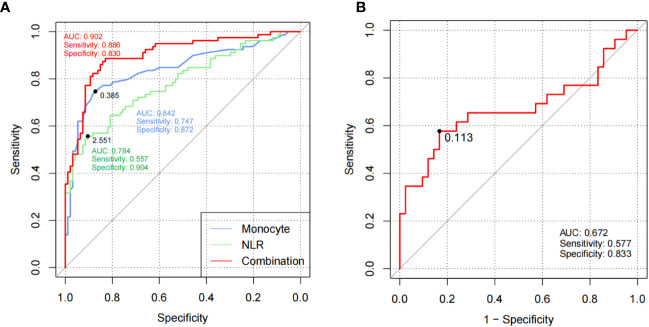
Models based on laboratory parameters were verified in cohort 2. **(A)** ROC curves of monocytes and NLR for RP versus HCs of replication cohort 2. **(B)** ROC curves of CAR for active RP versus inactive RP of replication cohort 2. The black plots in the curves represented the cutoff values. RP, relapsing polychondritis; NLR, neutrophil to lymphocyte ratio; HCs, healthy controls; ROC, receiver operating characteristic; AUC, area under the ROC curve.

The verification of the RP diagnosis model based on monocyte counts and NLR was conducted in cohort 2. The AUC, sensitivity, and specificity of monocyte counts (cutoff value = 0.385 × 10^9^/L) were 0.842, 74.7%, and 87.2% and those of NLR (cutoff value = 2.551) were 0.784, 55.7%, and 90.4%, respectively. After combined application, the diagnostic performance increased to 0.902, 88.6%, and 83%, respectively (**Figure 2A**), which indicated that the laboratory parameter models are potential indices to predict the occurrence of RP.

CAR also exhibited good clinical efficiency in activity assessment with an AUC of 0.672, sensitivity of 57.7%, and specificity of 83.3% (**Figure 2B**), which demonstrated that CAR could serve as a useful indicator to identify active RP patients.

Furthermore, a diagnostic nomogram including monocyte counts and NLR and an activity-monitoring nomogram enrolling CAR were developed using cohort 1 and 2 data, which could help predict the risk of RP occurrence and flare (**Supplementary Figure 1**).

### Lymphocyte subset changes in patients with RP

**Figure 3:**
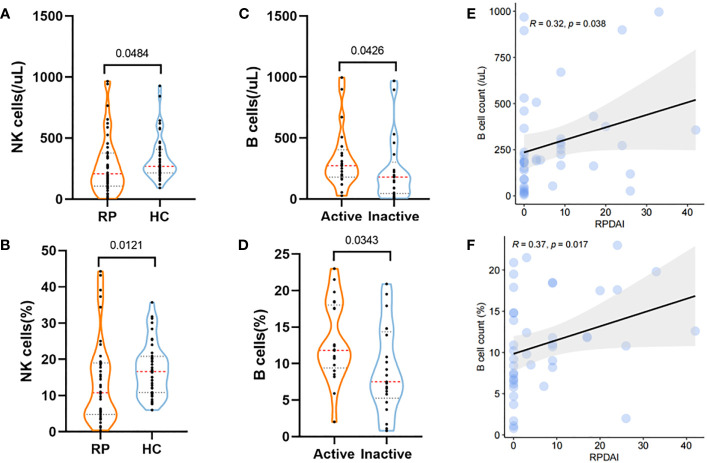
Lymphocyte subset changes in RP and their correlations with disease activity. **(A, B)** The levels of counts **(A)** and percentage **(B)** of NK cells in RP patients and HCs. **(C, D)** Violin plots showing the levels of B-cell counts **(C)** and percentage **(D)** in RP patients at the active stage and inactive stage. **(E, F)** The correlations of B-cell counts **(E)** and percentage **(F)** with the RPDAI score in RP patients. RP, relapsing polychondritis; HCs, healthy controls; NK cell, natural killer cell; RPDAI, Relapsing Polychondritis Disease Activity Index.

Lymphocyte cells play an important role in the pathological progress of RP. In total, 42 RP patients and 42 HCs from cohort 1 and cohort 2 were analyzed for the changes in lymphocyte subsets. Compared with HCs, RP patients showed a significant decrease in both the count and percentage of NK cells (*p* = 0.048 and *p* = 0.012, respectively) (**Figures 3A, B**, **Supplementary Table 6**).

In addition, active RP patients exhibited higher levels of B-cell count and percentage (*p* = 0.034 and *p* = 0.043, respectively) than patients with inactive RP (**Figures 3C, D**), which were also associated with the RPDAI score (**Figures 3E, F**).

## Discussion

Over the years, many biomarkers have been proposed to facilitate the diagnosis and prognosis of RP (7–9, 19). Nevertheless, none has been actually used in routine clinical practice. In the present study, we developed two laboratory parameter models, namely, monocyte counts combined with NLR, and CAR, to use in the diagnosis and recurrence monitoring of RP and identified lower NK cell levels in RP patients and higher B-cell levels in active patients. This might increase the existing knowledge of RP.

Various inflammatory-related markers, including WBC, neutrophils, monocytes, CRP, and others, were found to be elevated in RP patients, consistent with its systemic inflammatory feature. This study first represents that the model including monocyte counts and NLR could recognize RP patients from HCs. This model exhibited good clinical utility with an AUC of 0.845 and 0.902, sensitivity of 0.654 and 0.886, and specificity of 0.894 and 0.830 in the discovery and replication cohorts, respectively. Immune cells were proven to participate in the pathological progress of RP. Biopsies of ear cartilage revealed the accumulation of lymphocytes, macrophages, neutrophils, and plasma cells in the perichondrium of patients with early RP (20, 21). Moreover, enhanced spontaneous neutrophil extracellular trap formation suggested heavy inflammation caused by activated neutrophils (22). Peripheral blood classical monocytes were also increased in RP patients (23). Serum monocyte chemotactic protein-1, macrophage migration inhibitory factor, macrophage inflammatory protein-1, and IL-8 levels, which are involved in regulating monocyte/macrophage function, were higher in RP patients than in HCs (24, 25), suggesting the critical roles of monocytes, neutrophils, and lymphocytes in the pathogenesis of RP.

Additionally, the alterations in lymphocytes may enhance our understanding of the pathophysiology of RP. NK cells were reduced in RP patients compared with HCs, which is similar to the finding of Takagi et al. that natural killer T cells were decreased in patients with RP (26). NK cells play critical roles in innate and adaptive immune responses. The reduction in peripheral NK cells is not conducive to the clearance of pathogenic CD4^+^T cells in SLE patients, leading to the progression of SLE (27). In the present study, B cells increased in active RP patients and were positively correlated with RPDAI. High titers of anti-cartilage and anti-type II collagen antibodies were observed in active patients (28, 29), demonstrating B-cell activation. The phenomenon of activated humoral immune response might aggravate the patient’s condition and induce RP recurrence. Future studies exploring the specific role of immune cell production and migration in RP are warranted.

The results revealed that laboratory markers, such as CRP, CAR, NLR, PLR, and C3, were positively correlated with RPDAI, and other markers, such as albumin, prealbumin, and hemoglobin, were negatively correlated with RPDAI. The one that interests us is C3, which might play a pathogenic role in RP. Shirota et al. observed an elevated serum complement (50% hemolytic unit of complement) level in an RP patient (30). The deposits of immunoglobulins and C3 at the chondrofibrous junction of ear biopsy specimens were demonstrated by direct immunofluorescence examination (31, 32). In addition, C5-delete mice were less likely to develop RP after matrilin-1 induction than C5-normal mice (33). These collective findings suggested that the complement system might be involved in the development of RP.

The positive association between CAR and the RPDAI score in this study is in agreement with a previous study (14). In discovery cohort 1, CAR could identify active RP patients from stable patients with an AUC of 0.758, sensitivity of 0.60, and specificity of 0.84. Notably, CAR has been the focus of a few studies. It was concluded as a marker of disease activity in Takayasu arteritis (34), an independent risk factor of mortality in acute pancreatitis (35), and an independent predictor of adverse 30-day outcomes in septic shock (36). The present study findings revealed and validated that in RP, CAR was a reliable laboratory parameter to discern patients with relapse or flare.

The current study has some limitations. First, most of the patients included in this study were not initial onset and had received medication, and the effect of medication on markers was not assessed. Second, this was a single-center study, and the number of patients included in the lymphocyte subset analysis was small. Future multicenter studies are needed to validate our results.

## Conclusion

This study demonstrates the utility of models based on laboratory parameters in the clinical practice of RP. Specifically, monocyte counts and NLR could assist in RP diagnosis, and CAR as an independent risk factor could predict disease activity. Changes in C3 and lymphocyte subsets were correlated with the RPDAI score and aligned with the pathological process of RP. These cost-effective laboratory markers can be easily popularized and used in the clinical management of RP.

## Data availability statement

The raw data supporting the conclusions of this article will be made available by the authors, without undue reservation.

## Ethics statement

The studies involving humans were approved by the Institutional Review Board of PUMCH with informed consents acquired from all enrolled participants (I-23PJ540). The studies were conducted in accordance with the local legislation and institutional requirements. The participants provided their written informed consent to participate in this study.

## Author contributions

YML: Conceptualization, Investigation, Visualization, Writing – original draft, Writing – review & editing. LC: Data curation, Supervision, Visualization, Writing – review & editing, Funding acquisition. MZ: Conceptualization, Supervision, Visualization, Writing – review & editing. HZ: Data curation, Investigation, Visualization, Writing – review & editing. XL: Data curation, Investigation, Writing – review & editing. YHu: Investigation, Methodology, Writing – review & editing. HL: Investigation, Visualization, Writing – review & editing. YHo: Project administration, Resources, Supervision, Writing – review & editing, Conceptualization, Investigation. YZL: Conceptualization, Funding acquisition, Project administration, Resources, Supervision, Writing – review & editing.

## Supplementary material

Supplementary Figure 1 Diagnostic and monitoring nomograms for RP based on identified laboratory indexes. **(A)**. The nomogram including monocyte counts and NLR for predicting the risk of RP occurrence (left); an example showed the risk of patient 3# diagnosed with RP (right). **(B)**. The nomogram of CAR in quantifying the risk RP flare (left); an example showed the risk of patient 7# at active stage (right). RP, relapsing polychondritis. NLR, neutrophil to lymphocyte ratio.

Supplementary Table 1 Demographic characteristics of participants included in this study.

Supplementary Table 2 Clinical characteristics of RP patients in this study.

Supplementary Table 3 Multivariate binary logistic regression analysis of clinical indicators between RP and HCs of cohort 1.

Supplementary Table 4 Correlation between RPDAI score and laboratory parameters in cohort 1.

Supplementary Table 5 Multivariate binary logistic regression analysis of laboratory parameters between RP patients at active and inactive stage in cohort 1.

Supplementary Table 6 Lymphocyte subsets characteristic of RP patients and HCs.
