# Supplementary figures and images for: Development and validation of diagnostic and activity-assessing models for relapsing polychondritis based on laboratory parameters
Source: Front Immunol. 2023 Oct 3;14:1274677. doi: 10.3389/fimmu.2023.1274677 (PMC10579920; doi:10.3389/fimmu.2023.1274677)

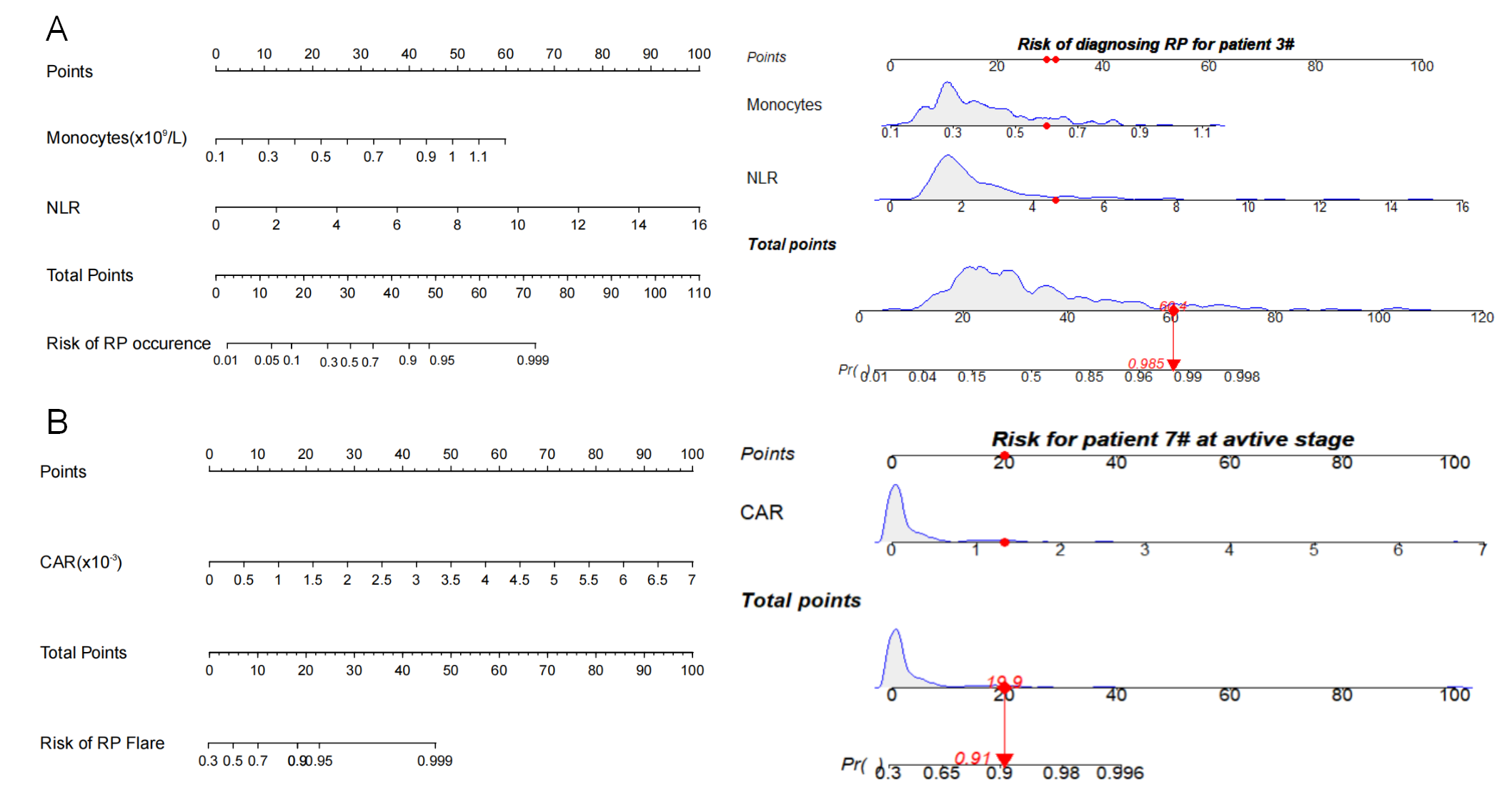

Supplement: Supplementary Figure 1 — Diagnostic and monitoring nomograms for RP based on identified laboratory indexes. (A). The nomogram including monocyte counts and NLR for predicting the risk of RP occurrence (left); an example showed the risk of patient 3# diagnosed with RP (right). (B). The nomogram of CAR in quantifying the risk RP flare (left); an example showed the risk of patient 7# at active stage (right). RP, relapsing polychondritis. NLR, neutrophil to lymphocyte ratio. [file Image_1.tif]
