# Supplementary material for: Development and validation of diagnostic and activity-assessing models for relapsing polychondritis based on laboratory parameters
Source: Front Immunol. 2023 Oct 3;14:1274677. doi: 10.3389/fimmu.2023.1274677 (PMC10579920; doi:10.3389/fimmu.2023.1274677)
Supplement: Supplementary Table 1 — Demographic characteristics of participants included in this study. [file Table_1.docx]

Supplementary Table 1 Demographic characteristics of participants included in this study

| **Characteristic** | **RP** | | | **HCs** | | |
| --- | --- | --- | --- | --- | --- | --- |
|  | **Total** | **Cohort 1** | **Cohort 2** | **Total** | **Cohort 1** | **Cohort 2** |
| Number, n | 157 | 78 | 79 | 188 | 94 | 94 |
| Gender (F/M) | 90/67 | 46/32 | 44/35 | 100/88 | 51/43 | 49/45 |
| Age, median (IQR), years | 49 (19) | 46 (17.5) | 51 (18) | 49 (19 | 47 (24) | 50 (5) |

Abbreviation: RP: Relapsing polychondritis; HCs: healthy controls. IQR: interquartile range, displaying as “Q3- Q1”.
