# Supplementary material for: Development and validation of diagnostic and activity-assessing models for relapsing polychondritis based on laboratory parameters
Source: Front Immunol. 2023 Oct 3;14:1274677. doi: 10.3389/fimmu.2023.1274677 (PMC10579920; doi:10.3389/fimmu.2023.1274677)
Supplement: Supplementary Table 2 — Clinical characteristics of RP patients in this study. [file Table_2.docx]

Supplementary Table 2 Clinical characteristics of RP patients in this study

| **Characteristics** | **Cohort 1** | | | **Cohort 2** | | |
| --- | --- | --- | --- | --- | --- | --- |
|  | **Total** | **Active RP** | **Inactive RP** | **Total** | **Active RP** | **Inactive RP** |
| Number, n | 78 | 35 | 43 | 79 | 28 | 51 |
| Age, mean (SD), years | 43.76 (13.76) | 43.91 (15.98) | 43.63 (11.84) | 49.16 (12.98) | 48.14 (14.38) | 49.90 (12.32) |
| Gender (F/M) | 46/32 | 18/17 | 28/15 | 44/35 | 18/10 | 26/25 |
| RPDAI score, mean (SD) | 9.05 (12.24) | 19.77 (11.13) | 0.33 (0.89) | 6.13 (9.81) | 17.29 (8.84) | 0 (0) |
| **Organ involvement, N (%)**^a^ | | | | | | |
| Respiratory tract | 54 (69.23) | 22(62.86) | 32(74.42) | 47(59.49) | 16(57.14) | 31(60.78) |
| Ears | 51 (65.38) | 26(74.29) | 25(58.14) | 48(60.76) | 20(71.43) | 28(54.90) |
| Nose | 46 (58.97) | 23(65.71) | 23(53.49) | 22(27.85) | 12(42.86) | 10(19.61) |
| Joints | 33 (42.31) | 18(51.43) | 15(34.88) | 14(17.72) | 6(21.43) | 8(15.69) |
| Sternocostal cartilage | 22 (28.21) | 10(28.57) | 12(27.91) | 9(11.39) | 5(17.86) | 4(7.84) |
| Eyes | 25 (32.05) | 14(40.00) | 11(25.58) | 19(24.05) | 8(28.57) | 11(21.57) |
| Nerve system | 7 (8.97) | 4(11.43) | 3(6.98) | 9(11.39) | 6(21.43) | 3(5.88) |
| **Treatments, n (%)^b^** | | | | | | |
| No treatments | 9(11.54) | 4(11.43) | 5(11.63) | 6(7.59) | 3(10.71) | 3(5.88) |
| GCs | 16(20.51) | 9(25.71) | 7(16.28) | 22(27.85) | 10(35.71) | 12(23.53) |
| Immunosuppressants ^c^ | 7(8.97) | 1(2.86) | 6(13.95) | 5(6.33) | 1(3.57) | 4(7.84) |
| Biologic agents/Small molecular targeted agents ^d^ | 0(0) | 0(0) | 0(0) | 0(0) | 0(0) | 0(0) |
| GCs and Immunosuppressants | 26(33.33) | 12(34.29) | 14(32.56) | 33(41.77) | 8(28.57) | 25(49.02) |
| GCs and Biologic agents/Small molecular targeted agents | 5(6.41) | 1(2.86) | 4(9.30) | 1(1.27) | 0(0) | 1(1.96) |
| Immunosuppressants and Biologic agents/Small molecular targeted agents | 0(0) | 0(0) | 0(0) | 0(0) | 0(0) | 0(0) |
| GCs and Immunosuppressants and Biologic agents/Small molecular targeted agents | 11(14.10) | 5(14.29) | 6(13.95) | 11(13.92) | 6(21.43) | 5(9.80) |
| Others ^e^ | 3(3.85) | 2(5.71) | 1(2.33) | 1(1.27) | 0(0) | 1(1.96) |

^a^ Organ involvements since onset are shown, RP patients had single organ lesion or multi-organ lesions.

^b^ The treatments of the patients with RP at the time of blood collection are shown.

^c^ Immunosuppressants include Methotrexate, Cyclosporine, Tacrolimus, Azathioprine, Cyclophosphamide, Leflunomide, Mycophenolate mofetil, Tripterygium glycosides.

^d^ Biologic agents/Small molecular targeted agents include Infliximab, Tocilizumab, Adalimumab, Etanercept, Tofacitinib, baricitinib.

^e^ Other drugs include Tripterygium Glycosides, antibiotics, Tobramycin and Dexamethasone Eye Drops, Bromfenac Sodium Eye Drops, and Ciclosporin Eye Drops.

Abbreviation: RP: Relapsing polychondritis; HCs: healthy controls. GCs: glucocorticoids.
