# Supplementary material for: Development and validation of diagnostic and activity-assessing models for relapsing polychondritis based on laboratory parameters
Source: Front Immunol. 2023 Oct 3;14:1274677. doi: 10.3389/fimmu.2023.1274677 (PMC10579920; doi:10.3389/fimmu.2023.1274677)
Supplement: Supplementary Table 3 — Multivariate binary logistic regression analysis of clinical indicators between RP and HCs of cohort 1. [file Table_3.docx]

Supplementary Table 3 Multivariate binary logistic regression analysis of clinical indicators between RP and HCs of cohort 1

| **Laboratory parameters** | **B** | **SE** | **Wald** | **OR (95%CI)** | ***p*** |
| --- | --- | --- | --- | --- | --- |
| Monocyte (×10^8^/L) | 0.588 | 0.213 | 7.623 | 1.800 (1.186-2.732) | 0.006 |
| Neutrophil (×10^9^/L) | 0.178 | 0.214 | 0.690 | 1.194(0.785-1.816) | 0.406 |
| Platelet (×10^11^/L) | 0.545 | 0.345 | 2.491 | 1.724(0.877-3.391) | 0.115 |
| Neutrophil to lymphocyte ratio | 0.839 | 0.310 | 7.342 | 2.314(1.261-4.246) | 0.007 |
