# Supplementary material for: Development and validation of diagnostic and activity-assessing models for relapsing polychondritis based on laboratory parameters
Source: Front Immunol. 2023 Oct 3;14:1274677. doi: 10.3389/fimmu.2023.1274677 (PMC10579920; doi:10.3389/fimmu.2023.1274677)
Supplement: Supplementary Table 4 — Correlation between RPDAI score and laboratory parameters in cohort 1. [file Table_4.docx]

Supplementary Table 4 Correlation between RPDAI score and laboratory parameters in cohort 1

| **laboratory indexes** | **RPDAI Score** | |
| --- | --- | --- |
|  | **r** | ***p*** |
| Erythrocyte sedimentation rate (mm/h) | 0.51 | <0.001 |
| C-reactive protein (mg/L) | 0.45 | <0.001 |
| C-reactive protein to albumin ratio (10^-3^) | 0.47 | <0.001 |
| Complement 3 (g/L) | 0.40 | 0.025 |
| Interleukin-6 (pg/ml) | 0.37 | 0.036 |
| White blood cell (×10^9^/L) | 0.26 | 0.024 |
| Neutrophil (%) | 0.29 | 0.009 |
| Neutrophil (×10^9^/L) | 0.28 | 0.012 |
| Lymphocyte (%) | -0.27 | 0.016 |
| Neutrophil to lymphocyte ratio | 0.29 | 0.011 |
| Platelet (×10^9^/L) | 0.39 | <0.001 |
| platelet to lymphocyte ratio | 0.30 | 0.008 |
| Albumin (g/L) | -0.53 | <0.001 |
| Albumin to globulin ratio | -0.49 | <0.001 |
| Basophil (%) | -0.19 | 0.004 |
