# Supplementary material for: Development and validation of diagnostic and activity-assessing models for relapsing polychondritis based on laboratory parameters
Source: Front Immunol. 2023 Oct 3;14:1274677. doi: 10.3389/fimmu.2023.1274677 (PMC10579920; doi:10.3389/fimmu.2023.1274677)
Supplement: Supplementary Table 5 — Multivariate binary logistic regression analysis of laboratory parameters between RP patients at active and inactive stage in cohort 1. [file Table_5.docx]

Supplementary Table 5 Multivariate binary logistic regression analysis of laboratory parameters between RP patients at active and inactive stage in cohort 1

| **Laboratory parameters** | **B** | **SE** | **Wald** | **OR（95% CI）** | ***p*** |
| --- | --- | --- | --- | --- | --- |
| C-reactive protein to albumin ratio (10^-3^) | 1.487 | 0.705 | 4.447 | 4.422(1.111-17.605) | **0.035** |
| Platelet to lymphocyte ratio (10^2^) | 0.279 | 0.317 | 0.773 | 1.322(0.710-2.460) | 0.379 |
