# Supplementary material for: Development and validation of diagnostic and activity-assessing models for relapsing polychondritis based on laboratory parameters
Source: Front Immunol. 2023 Oct 3;14:1274677. doi: 10.3389/fimmu.2023.1274677 (PMC10579920; doi:10.3389/fimmu.2023.1274677)
Supplement: Supplementary Table 6 — Lymphocyte subsets characteristic of RP patients and HCs. [file Table_6.docx]

Supplementary Table 6 Lymphocyte subsets characteristic of RP patients and HCs

|  | **RP**  **( n=42)** | **HCs**  **( n=42)** | ***p* value** | **Active RP**  **( n=21)** | **Inactive RP ( n=21)** | ***p* value** |
| --- | --- | --- | --- | --- | --- | --- |
| Lymphocyte (×10^9^/L)^a^ | 2.15(1.48) | 1.84(0.49) | 0.253 | 2.35(1.03) | 2.14(1.26) | 0.561^a^ |
| B cells (%) | 1075(8.73) | 10.05(3.25) | 0.829 | 12.99(5.38) | 9.18(5.88) | **0.043**^a^ |
| B cells (/ul) | 197.5(256.7) | 179.5(74.5) | 0.348 | 273(225) | 179(257) | **0.034** |
| NK cells (%) | 10.75(14.23) | 10.6(10) | **0.012** | 14.5(13.85) | 9.7(14.75) | 0315 |
| NK cells (/ul) | 206.5(271.30) | 267.5(193.5) | **0.048** | 336(388.5) | 195(174.5) | 0.176 |
| T cells (%) | 74.48(12.18) | 71.34(7.96) | 0.166^a^ | 72.08(9.34) | 76.88(14.32) | 0.205^a^ |
| T cells (/ul) | 1599(1245.5) | 1338(391) | 0.255 | 1674(724.7) | 1651(1038) | 0.933^a^ |
| T4 cells (%)^a^ | 39.94(12.94) | 39.1(7.16) | 0.715^a^ | 41.12(12.48) | 38.75(13.58) | 0.558^a^ |
| T4 cells (/ul) | 805.5(716) | 728(275.8) | 0.409 | 825(518.5) | 712(1101) | 05416 |
| T8 cells (%) | 26.8(15.65) | 24.55(9) | 0.324 | 23.8(15.1) | 30.1(21.95) | 0.118 |
| T8 cells (/ul) | 548(421.8) | 491.5(192.8) | 0.116 | 580.1(242.7) | 657.9(394.2) | 0.446^a^ |
| T4/T8 | 1.36(1.31)^a^ | 1.56(0.81)^a^ | 0.506 | 1.85(1.06) | 1.44(0.90) | 0.189^a^ |

^a^ represents normally distributed data, expressed as mean(SD). Others are non-normally distributed data, expressed as median(IQR), IQR=Q3-Q1. Abbreviation: RP, Relapsing polychondritis. HCs, healthy controls. NK cells, natural killer cells; T4/T8, CD4^+^T to CD8^+^T cell ratio.
